# ﻿*Chimonobambusafarcta* (Poaceae, Bambusoideae), a new species from western Guangxi, China with taxonomic notes on *C.pubescens* and *C.luzhiensis*

**DOI:** 10.3897/phytokeys.239.116592

**Published:** 2024-03-05

**Authors:** Guang-Hui Lai, Jin-Jun Yue

**Affiliations:** 1 Guangde Forestry Bureau of Anhui Province, 242200, Guangde, China Guangde Forestry Bureau of Anhui Province Guangde China; 2 Research Institute of Subtropical Forestry, Chinese Academy of Forestry, 311400, Hangzhou, China Research Institute of Subtropical Forestry, Chinese Academy of Forestry Hangzhou China

**Keywords:** Amphipodial rhizome, Arundinarieae, taxonomy, temperate woody bamboos

## Abstract

*Chimonobambusafarcta*, a new species of temperate woody bamboos from western Guangxi, China is described and illustrated. The new species is similar to *C.pubescens* in the solid internodes of culms, but differs in having taller culm to 7 m with longer verrucose internodes to 23.5 cm and intranodes to 4 mm, intranode usually with a ring of 7–9 root thorns below mid-culm, abaxially brown or brown-purple verrucose-setose culm leaf sheaths with indistinct transverse veins, conspicuously developed culm leaf blades to 3.2 cm long, longer foliage leaf sheaths to 5.2 cm, larger and broader foliage leaf blades to 22 × 1.4 cm. It also somewhat resembles *C.convoluta*, but can be easily distinguished by having solid internodes and longer intranode 2–4 mm, very prominent nodes with supranodal ridge obviously more elevated than sheath scar, usually persistent and sometimes brownish striate culm leaf sheaths, longer culm leaf blades to 3.2 cm, and abaxially glabrous foliage leaf sheaths. Based on the morphological characteristics, this new species is assigned to C.sect.Chimonobambusa. The character description of *C.pubescens* are revised for its culm to 2.1 m tall, 1.1 cm in diameter and glabrous foliage leaf blades. The systematic position of *C.luzhiensis* is discussed, and this species is proposed as a member of C.sect.Chimonobambusa.

## ﻿Introduction

*Chimonobambusa* Makino (s. l., including *Qiongzhuea* Hsueh & Yi), as a relatively large genus in Bambusoideae, is distributed from the south part of East Asia through the middle and north parts of Southeast Asia to the north part of South Asia subcontinent, with a distribution center around the Yunnan-Guizhou Plateau and Sichuan Basin (Hsueh and Zhang 1988; Wen 1994; Xue and Zhang 1996). So far, 69 Latin binomens were published or transferred under this genus (urn:lsid:ipni.org:names:17734-1), of which about 38 species were recognized by several works (Wen 1994; Ohrnberger 1999; Li and Stapleton 2006; Naithani 2009), with 25 species in the distribution center. Only one species spreads eastward to the south of central Honshu, Japan, and a few species reach southward to northern Vietnam, Laos, northern Myanmar, and westward to Tibet, China and northern India (Wen 1994; Ohrnberger 1999). According to Huang and Dai (2009), there are five species of *Chimonobambusa* in Guangxi, a provincial administrative region located in the south of Chinese Mainland and near the modern distribution center of this genus. Later, Xia et al. (2016) recorded six species, including a cultivated species in "*Flora of Guangxi Vol.5*". Compared with Yunnan (Li and Xue 2003), Guizhou (Lan 1988) and Sichuan (Yi 1997), although the plants of this genus in Guangxi are not abundant, two species, *C.damingshanensis* Hsueh & W. P. Zhang and *C.convoluta* Q. H. Dai & X. L. Tao, are endemic to the region. In recent years, the investigation and taxonomy of this genus are still continuing, and some taxa were reported as new species or transferred into this genus (Cao et al. 2022; Niu et al. 2022). The economic value of plants in this genus is relatively high. Generally, the bamboo shoots taste delicious and can be used for fresh food or making pickled and dried shoots. Many species have high ornamental value for their more or less square culms, root thorns in the intranodes, and narrow and dense leaves, and are used for gardening, landscaping or ornamentation in urban and rural greening. Some species grow on steep hillsides or under broadleaved forests, which are also good plants for soil and water conservation.

During the introduction process of building Guangde National Bamboo Germplasm Resource Bank, we made some investigations on the scattered bamboo species distributed in the subtropical zone of China. In early November 2022, on the way back to the west of Lingyun County in western Guangxi after a special expedition to *Sinobambusa* Makino ex Nakai, a shrubby bamboo forest on the steep hillside at the roadside drew our attention. At that time, it was in the late stage of shooting, and its characteristics, including scattered culms, intranodes with developed root thorns, persistent culm leaves, underdeveloped culm leaf blades, and narrow foliage leaf blades clearly indicate that it belongs to *Chimonobambusa*. The internodes of both new and old culms of this unknown bamboo are solid, which is similar to that of *Chimonobambusapubescens* Wen. However, it can be readily distinguished from the latter by having taller culm, longer verrucose internodes, longer intranodes with developed root thorns, abaxially brown or brown-purple verrucose-hispid culm leaf sheath and relatively developed culm leaf blade. It also somewhat resembles *C.convoluta* Q. H. Dai & X. L. Tao, but obviously differs in having culm with solid internodes, very elevated nodes and usually persistent culm leaves. On the basis of further investigations and morphological comparison, it was identified as a new species different from all the known species of the genus *Chimonobambusa*, and is described and illustrated here.

## ﻿Materials and methods

The specimens of the new species were collected from Lingyun County in western Guangxi on 5 November 2022, and supplementary investigations and collections were separately made on 24 May 2023 and 22 October 2023. The morphological characteristics were observed and recorded from living materials, and some quantitative traits with taxonomic value were measured with a folding ruler rod and a vernier caliper. Specimens of the new species were deposited in AAUF, the herbarium of Guangde Forestry Institute, Anhui Province, China, the herbarium of Research Institute of Subtropical Forestry, Chinese Academy of Forestry and Anji Bamboo Exposition Garden, Zhejiang, China. Herbarium acronyms follow Thiers (2023). The terminology follows Soderstrom and Calderón (1978), Xue and Zhang (1996), Clark and Cortés (2004), Li and Stapleton (2006) and Qin et al. (2021).

## ﻿Taxonomy

### ﻿ Chimonobambusa farcta

Taxon classificationPlantaePoalesPoaceae

G.H.Lai & J.J.Yue sp. nov.

0FB73D1B-836C-57D8-B060-22D9DD9E51AB

urn:lsid:ipni.org:names:77314616-1

Figs 1 , 2 , 3

#### Diagnosis.

*Chimonobambusafarcta* with solid internodes of culms is similar to *C.pubescens*, but differs in having taller culm to 7 m (vs. 2.1 m) with longer internodes to 23.5 cm (vs. 14 cm) and intranodes to 4 mm (vs. 2 mm), intranode usually with a ring of 7–9 root thorns below mid-culm, abaxially brown or brown-purple verrucose-setose culm leaf sheaths with indistinct transverse veins, conspicuously developed culm leaf blade to 32 mm (vs. ca. 2 mm)long, longer foliage leaf sheaths to 5.2 cm (vs. 2.8 cm), larger and broader foliage leaf blade to 22 × 1.4 (vs. 12 × 0.9 cm) cm. It also somewhat resembles *C.convoluta*, but can be easily distinguished by having culm with solid internodes and longer intranode 2–4 mm (vs. 1–2 mm), very prominent nodes with supranodal ridge obviously more elevated than sheath scar, usually persistent and sometimes brownish striate culm leaf sheaths, longer culm leaf blade to 32 mm (vs. 20 mm), and abaxially glabrous foliage leaf sheaths (Table 1).

**Table 1.** Morphological comparison of *Chimonobambusafarcta* and its related species.

Character	*Chimonobambusafarcta*	*Chimonobambusapubescens*	*Chimonobambusaconvoluta*
Culm height	3–7 m	0.7–2.1 m	2–3 m
Culm internode	12–23.5 cm long, solid, basically weakly flattened on branch-bearing side, with retrorse white hairs arranged in longitudinal rows and curved verrucae	(5–) 8–14 cm long, solid, flattened on branch-bearing side, initially white pubescent, without verrucae	12–20 cm long, hollow, grooved on branch-bearing side, initially sparsely fulvous verrucose-hispid and becoming verrucose after setae falling
Culm intranode	2–4 mm long, usually with a ring of 7–9 root thorns at nodes below mid-culm	2 mm long, with some short aerial roots at nodes of basal culm	1–2 mm long, usually with a ring of 5–7 root thorns at nodes below mid-culm
Culm node	very prominent, supranodal ridge obviously more elevated than sheath scar	prominent, supranodal ridge more elevated than sheath scar	moderately prominent, supranodal ridge slightly more elevated than or equaling to sheath scar
Culm leaf	usually persistent	usually persistent	deciduous
Culm leaf sheath	with dense small purple-brown spots, sometimes brownish stirate, abaxially sparsely brown or brown-purple setose, with indistinct transverse veins	unspotted, abaxially mainly glabrous, with distinct transverse veins	with small purple-brown spots, abaxially sparsely brown setose, with indistinct transverse veins
Culm leaf fimbriae	absent, sometimes 1or 2 on each shoulder	absent	absent, sometimes 1or 2 on each shoulder
Culm leaf ligule	shorter than 1 mm tall, truncate or slightly arched,	ca. 2 mm tall, convex	shorter than 1 mm tall, slightly arched
Culm leaf blade length	4–32 mm	ca. 2 mm	11–20 mm
Foliage leaf	(2–)3–6(–9) per ultimate branchlet	3 or 4 per ultimate branchlet	2–4 per ultimate branchlet
Foliage leaf sheath	2.8–5.2 cm long, abaxially glabrous	2.5–2.8 cm long, abaxially glabrous	2.5–4.5 cm long, abaxially densely brown tomentose
Foliage leaf fimbriae	5–10 (–12) on each shoulder, 3–8 mm long	a few on each shoulder, 4–8 mm long	5–9 on each shoulder, 8–10 mm long
Foliage leaf blade	linear-lanceolate, 9–22 × 0.7–1.4 cm, secondary veins 3–6(–7)-paired	linear-lanceolate, 9–12 × 0.7–0.9 cm, secondary veins 3–5-paired	lanceolate, 16–22 ×1–1.5 cm, secondary veins 4- or 5-paired
Habitat and distribution	under evergreen broadleaved forests at the elevation of 1200–1500 m, Lingyun, western Guangxi	along the stream or under coniferous forests and thickets at the elevation of 350–800 m, southwestern Hunan	under broadleaved forests at the elevation of 800–1400 m, Tianlin and Napo, western Guangxi

**Figure 1.**
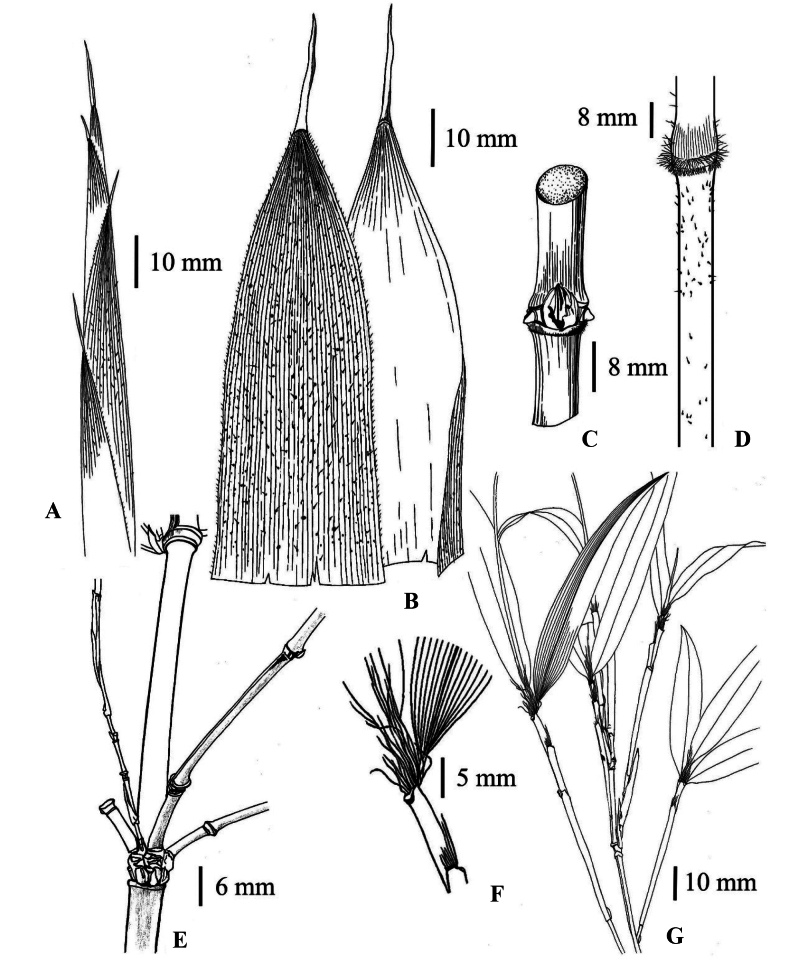
*Chimonobambusafarcta***A** upper part of shoot **B** culm leaf in abaxial view, showing sparse verrucose setae; culm leaf in adaxial view, showing slightly arched ligule and narrowly linear-lanceolate blade **C** part of culm, showing conical root thorns and narrowly ovate buds at node, and solid internode **D** base of culm leaf sheath and upper part of an internode of young culm, showing indumentum and curved verrucae **E** a node of mid-culm, showing branch complement **F** mouth of foliage leaf sheaths, showing developed fimbriae **G** branchlets and ultimate branchlets, showing foliage leaf complement. Illustrated by Sai-Jun Ma based on *G. H. Lai & J. J. Yue 22111*.

#### Type.

China. Guangxi: Lingyun County, Sicheng Town, Jinbao Village, Dashipo, 24°25'22"N, 106°30'29"E, 1261 m alt., 5 November 2022, *G. H. Lai & J. J. Yue 22111* (holotype: AAUF!; isotypes: herbarium of Guangde Forestry Institute! and herbarium of Research Institute of Subtropical Forestry, Chinese Academy of Forestry!).

**Figure 2.**
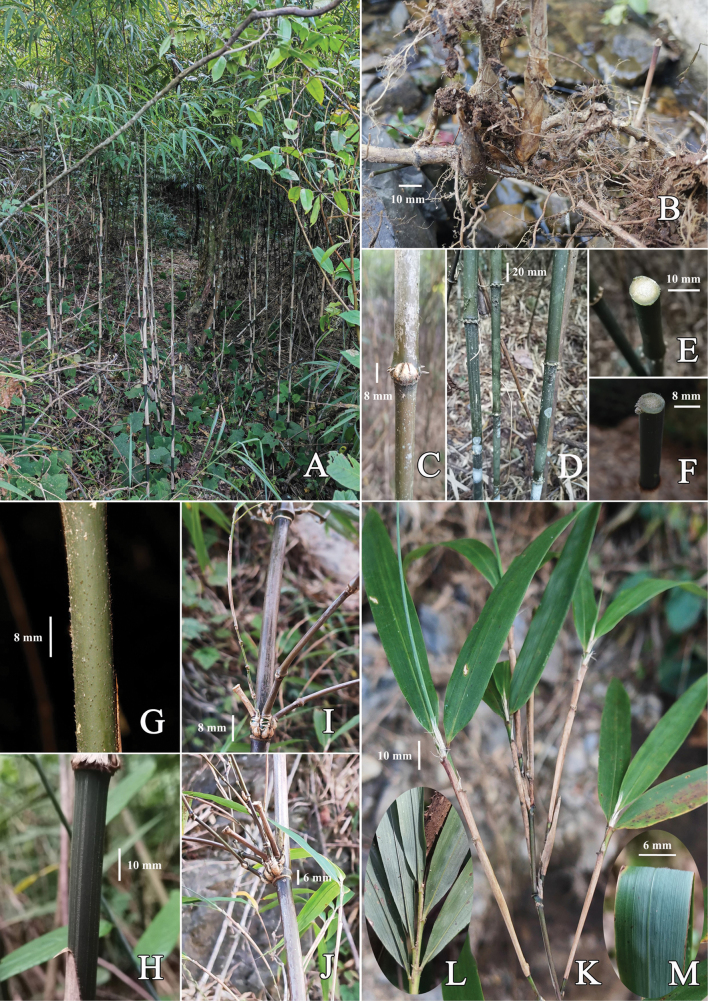
*Chimonobambusafarcta***A** clump **B** rhizome **C** a node in lower culm, showing bud complement **D** intranodes of lower culms, showing an arrangement of conical or top-shaped, hard root thorns **E, F** cross section of old and young culms, showing solid internode **G** part of culm internode, showing dense curved verrucae **H** upper part of an internode of young culm, showing short, retrorse hairs arranged in longitudinal rows **I, J** node of mid-culm or upper culm, showing branch complement **K** branchlet and foliage leaves in adaxial view, showing sheaths, fimbriae, and blades **L** base of foliage leaves in abaxial view **M** part of foliage leaves in abaxial view, showing pubescent abaxial surface. Photos by Guang-Hui Lai.

#### Description.

Shrubby bamboo. Rhizome amphipodial. Culms 3–7 m tall, 0.5–1.5 cm in diameter, erect, straight or sometimes middle and lower part of culm slightly zigzag and more or less oblique at nodes; internodes 12–23.5 cm long, cylindrical, basically weakly flattened on branch-bearing side, hard, solid (rarely subsolid on upper culm), dark green (rarely purple striate) and not pruinose when young, with white or pale brown curved verrucae (more on bare part) and white short retrorse hairs arranged in longitudinal rows, green or brown when old, scarred and rough after verrucae falling, glabrescent, obviously dirty-powdery; intranode 2–4 mm long, usually with a ring of 7–9 hard root thorns below mid-culm; thorns top-shaped or conical, 2–5 mm long, horizontally spreading or slanted downward; supranodal ridge very prominent, sometimes geniculate-swollen on bud-bearing opposite side, obviously more elevated than sheath scar; sheath scars prominent, densely persistently brown setoses; buds 3 at each node, adnate, narrowly ovate or conical, middle one longer, prophyll ovate or broadly ovate, abaxially glabrous, margin brownish ciliate; branches initially 3 per node, later to more than 10 on upper nodes of culm, spreading. Culm leaves usually persistent, rarely late deciduous, obviously shorter than internodes (1/2–2/3 as long as them) on lower culm, subequal in length to, or longer than internodes on middle and upper culm; sheaths papery, variable in color, initially brownish, yellowish-green, yellowish-brown or purplish-brown, tinged with green and brownish-red toward convex apex, soon straw-colored, with small and dense purple-brown spots, sometimes brownish stirate, abaxially sparsely uniformly upward appressed brown or brown-purple verrucose-setose, long brown hispid near base, densely brown bristly at bottom together with sheath scar, marginally densely brown ciliate, obviously longitudinally ribbed, indistinctly transversely veined; auricles absent, fimbriae not developed, sometimes 1or 2 on each shoulder, suberect; ligule shorter than 1 mm, purple-brown, truncate or slightly arched, margin extremely shortly ciliolate or subglabrous; blade narrowly triangular, subulate or narrowly linear-lanceolate, initially green or brownish-green, tinged with yellow toward apex, 4–32 × 1.5–2.5 mm, erect, not articulate with sheath. Foliage leaves (2–)3–6(–9) per ultimate branchlet; sheaths 2.8–5.2 cm long, abaxially glabrous, margins white or yellowish ciliolate sometimes hardly ciliate; auricles inconspicuous, fimbriae developed, 5–10(–12) on each shoulder, 3–8 mm long, grey-white, neatly arranged, straightly extended, easily deciduous or broken; ligule not exserted, shorter than 1 mm, truncate, apex hardly or very shortly ciliolate; petiole 1–2 mm long, glabrous; blade linear-lanceolate, 9–22 × 0.7–1.4 cm, adaxially green and glabrous, abaxially greenish and white pubescent, secondary veins 3–6(–7)-paired, transverse veins distinct, apex long acuminate, base cuneate, one margin minutely serrulate-scabrid. Inflorescence and caryopsis unknown.

#### Vernacular names.

凌云寒竹(Chinese name), líng yún hán zhú (Chinese Pinyin); 实竹(Local common name), shí zhú (Chinese Pinyin).

#### Distribution and habitat.

**Figure 3.**
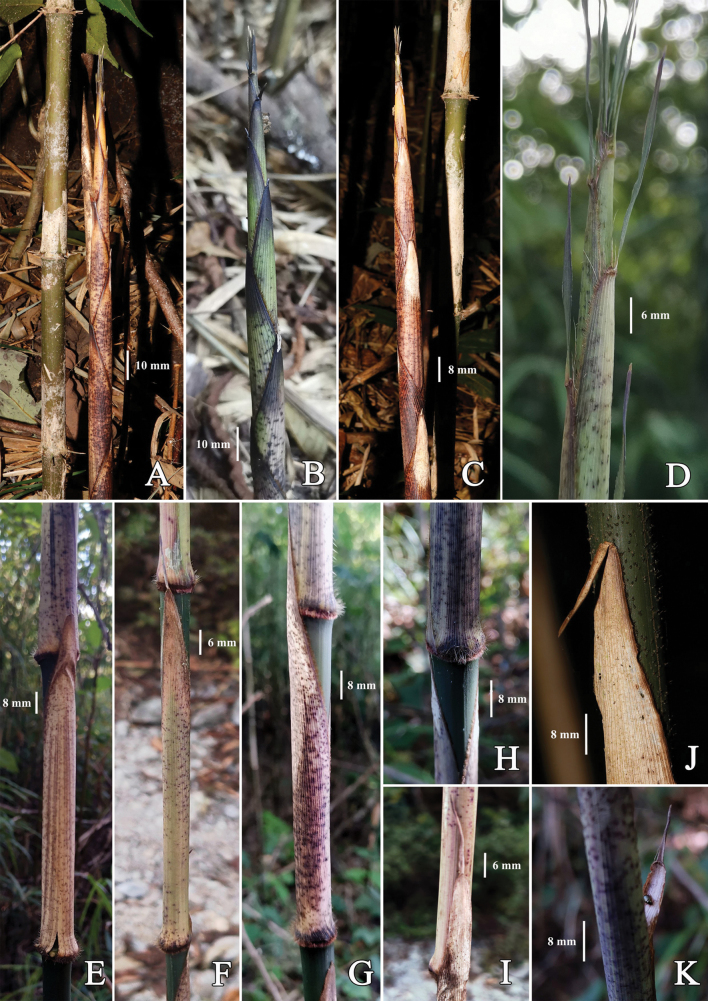
*Chimonobambusafarcta***A, B, C** shoots, showing variable colors on sheaths **D** upper part of shoot, showing a few fimbriae and nearly erect blade **E, F** culm leaf sheath in abaxial view, with small purple-brown spots and sparse verrucose setae, sometimes with brownish stripes **G** culm leaf sheath in lateral view, showing densely brown ciliate margins **H** base of culm leaf sheath, showing indumentum **I** upper part of culm leaf in abaxial view, showing arched top of sheath **J** upper part of culm leaf in abaxial view, showing truncate short ligule and blade **K** upper part of culm leaf in adaxial view, showing slightly arched short ligule. Photos by Guang-Hui Lai.

**Figure 4.**
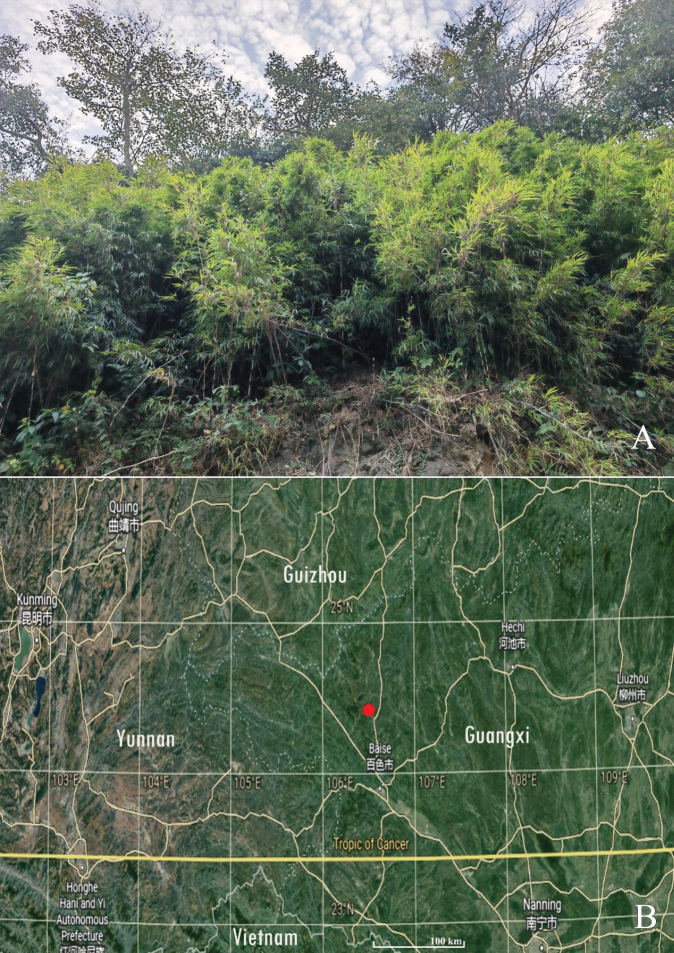
The habitat and geographical distribution of *Chimonobambusafarcta***A** clump growing in natural habitat **B** the red pentagon showing its distribution area. Photo A by Guang-Hui Lai.

*Chimonobambusafarcta* was only found from Lingyun County, between Mt. Qinglong and Mt. Donglan, western Guangxi, China. It grows under the evergreen broadleaved forest of steep hillsides at an altitude of 1200–1500 m (Figs 3, 4).

#### Conservation status.

This new species has been commonly found in the northwestern mountainous area of Lingyun County with quite a few populations, where its area of occupancy is less than 50 km^2^. Fortunately, its current distribution area happens to be included in the Sishuihe Nature Reserve and is under effective protection. Thus, it is assigned the status of “Least Concern” (LC) according to the IUCN Red List Categories and Criteria (IUCN Standards and Petitions Committee 2022).

#### Phenology.

New shoots developed in October.

#### Etymology.

The specific epithet “*farcta*” refers to the culms of the new species with solid internodes.

#### Additional specimens examined (paratypes).

**China. Guangxi**: Lingyun County, Sicheng Town, Jinbao Village, Dashipo, 24°25'52"N, 106°30'30"E, 1294 m alt., 5 November 2022, *J. X. Ma & D. D. Zhao 22001* (Anji Bamboo Exposition Garden); same locality, 24 May 2023, *G. H. Lai & J. J. Yue 23030* (herbarium of Guangde Forestry Institute); Lingyun County, Sicheng Town, Jinbao Village, Bajiaoshan, 24°24'51"N, 106°30'17"E, 1316 m alt., 22 October 2023, *G. H. Lai & J. J. Yue 23042* (herbarium of Guangde Forestry Institute).

## ﻿Discussion

*Chimonobambusa* Makino (s. l.) was divided into three sections by Wen and Ohrnberger (Ohrnberger 1990), namely C.sect.Chimonobambusa, C.sect.Oreocalamus (Keng) T.H.Wen & Ohrnb. and C.sect.Qiongzhuea (Hsueh & Yi) T.H.Wen & Ohrnb. They actually originated from three prior genera, *Chimonobambusa* Makino, 1914, *Oreocalamus* Keng, 1940 and *Qiongzhuea* Hsueh & Yi, 1980. Subsequently, Wen (1994) followed this treatment in his monographic study on the genus *Chimonobambusa* with 5 species under C.sect.Chimonobambusa. Xue and Zhang (1996) partially adopted this treatment in "*Flora Reipublicae Popularis Sinicae Tomus 9 (1)*", and Xue and Yi (1996) restored C.sect.Qiongzhuea to a separate genus *Qiongzhuea* whose shoots are not black under the action of enzymes, but C.sect.Chimonobambusa and C.sect.Oreocalamus were reserved in *Chimonobambusa*. Later, Stapleton and Xia (2004) pointed out the illegality of the generic name *Qiongzhuea* Hsueh & Yi, 1980 because its type species had not been effectively published, and regarded that *Qiongzhuea* was legally established in 1983 according to a *descriptio generico-specifico* with the type species *Chimonobambusaszechuanensis* (Rendle) Keng f., but its morphological characteristics were the same as those of the C.sect.Oreocalamus. Therefore, Chimonobambusasect.Qiongzhuea were treated as a synonym of C.sect.Oreocalamus, and most of the species described under the former were transferred into the latter, only two swollen node species were separated to establish a new group C.sect.Neoqiongzhuea Stapleton & N. H. Xia. Li and Stapleton (2006) recognized *Chimonobambusa* (s. l.) in "*Flora of China Vol. 22*", but no infrageneric group was defined. Both Yi et al. (2008) and Shi et al (2021) agree with the generic concept of Xue and Zhang (1996), but did not recognize the division of sections. We believe that there are about 9 species similar to the type species of *Chimonobambusa*, i.e. *C.marmorea*, with distinctive features in having persistent culm leaves and thinly papery or rarely papery culm leaf sheaths and constituted a relatively natural group. It is appropriate to place these species into C.sect.Chimonobambusa because they are obviously different from other bamboo species with deciduous culm leaves, papery or thickly papery culm leaf sheaths in the genus *Chimonobambusa* (s. l.). Some key vegetative characteristics of this new species, such as its slender and shorter culms with persistent culm leaves and very thin culm leaf sheaths, and new shoots developed in October, are rather consistent with those of C.sect.Chimonobambusa. Thus, this new species should be placed in this section. A key to the species of C.sect.Chimonobambusa is provided.

### ﻿Key to the species of Chimonobambusasect.Chimonobambusa

**Table:** 

1a	Culm leaf blades on middle and upper part of culm well-developed, longer than 10 mm	**2a**
2a	Culm intranodes without root thorns, initially glabrous	***Chimonobambusasichuanensis***
2b	Culm basal to middle intranodes with root thorns, initially pubescent	**3a**
3a	Culm internodes solid, verrucose; intranode 3–4 mm long; foliage leaf usually 3–6 per ultimate branchlet, foliage leaf blades 0.7–1.4 cm wide	***Chimonobambusafarcta***
3b	Culm internodes hollow, verrucose-hispid; intranode 1–2 mm long; foliage leaf usually 1 or 2 per ultimate branchlet, foliage leaf blades usually 1.4–2.5 cm wide	***Chimonobambusaleishanensis***
1b	Culm leaf blades on middle and upper part of culm slightly developed, shorter than 9 mm	**4a**
4a	Culm leaves shorter than internodes; culm leaf sheaths abaxially unmarked	**5a**
5a	Culm 0.7–2.1 m tall; internodes solid, pubescent; culm leaf blade ca. 2 mm long; foliage leaf blades 9–12 × 0.7–0.9 cm	***Chimonobambusapubescens***
5b	Culm 2.5–5 m tall; internodes hollow, glabrous; culm leaf blade 2–9 mm long; foliage leaf blades 15–23 × 1.6–2 cm	***Chimonobambusaluzhiensis***
4b	Culm leaves longer than internodes; culm leaf sheaths abaxially grey or brown spotted	**6a**
6a	Culm internodes initially glabrous; culm leaf sheaths abaxially grey spotted, glabrous or sparsely setose	**7a**
7a	Culm 1–1.5(–3) m tall, only basal intranodes with aerial roots or root thorns, culms cylindrical	***Chimonobambusamarmorea***
7b	Culm taller than 3 m, basal to middle intranodes with root thorns, culms slightly 4-angular	***Chimonobambusapurpurea***
6b	Culm internodes initially white pubescent; culm leaf sheaths abaxially brown spotted, densely setose	**8a**
8a	Culm internodes 10–13 cm long, hollow; culm leaf blade 3–4 mm long; foliage leaf ligule ca. 6 mm long	***Chimonobambusadamingshanensis***
8b	Culm internodes 7–8 cm long, subsolid; culm leaf blade shorter than 1 mm; foliage leaf ligule shorter than 1 mm long	***Chimonobambusabrevinoda***

**Figure 5.**
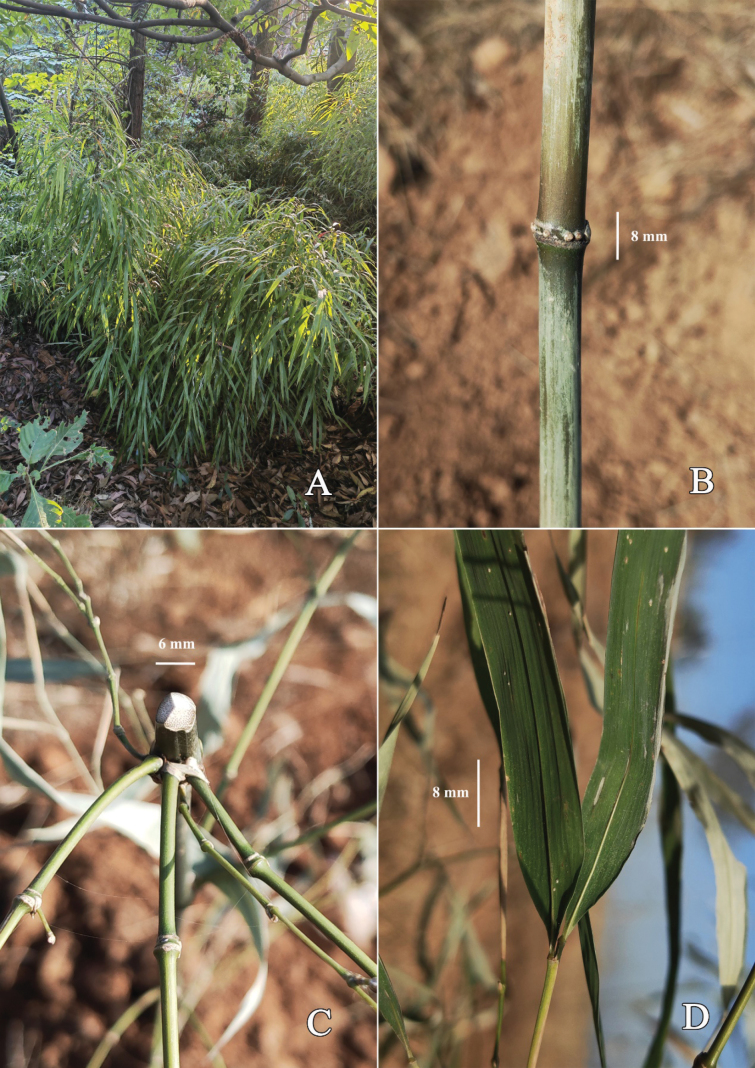
*Chimonobambusapubescens***A** clump **B** intranode of lower culm, showing an arrangement of short root thorns **C** cross section of old culm, showing solid internode **D** part of foliage leaves in abaxial view, showing glabrous abaxial surface. Photos by Guang-Hui Lai.

**Figure 6.**
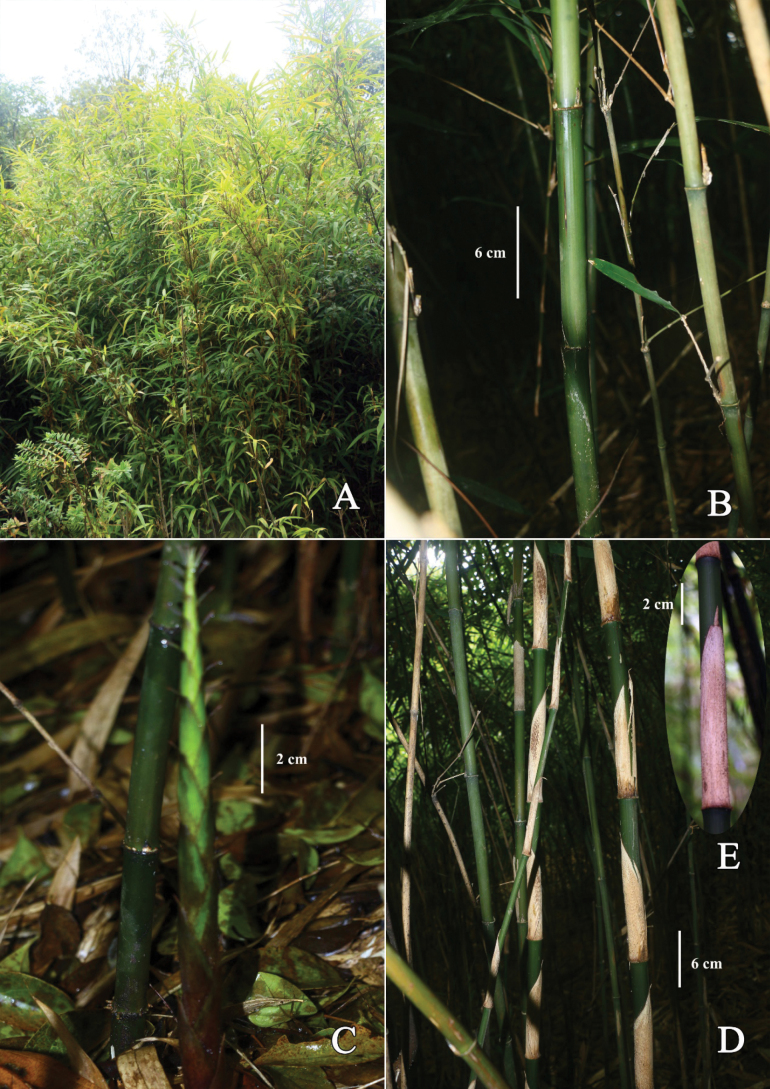
*Chimonobambusaluzhiensis***A** clump **B** a part of culms, showing cylindrical internodes **C** shoot and base of culm, showing nodes with protuberances or short aerial roots **D, E** persistent culm leaves. Photos by Guang-Hui Lai.

We also found that a vegetative description of *Chimonobambusapubescens* Wen is incomplete. In the protologue, Wen (1986) stated that the abaxial surface of foliage leaf blade was covered with silky hair in the diagnosis part, while he mentioned that both surfaces of foliage leaf blades were glabrous in the description part. Obviously, the description is inconsistent. In fact, both surfaces of foliage leaf blades of this species are glabrous (Fig. 5). *Chimonobambusasolida* B. M. Yang & C. Y. Zhang, which is morphologically very similar to *C.pubescens*, was described by Yang (1988) based on the specimens collected from Yiyang Forestry Institute where the propagating materials were introduced from Zhijiang County of Hunan Province. During our investigation on scattered bamboos from this province in 2022, we discovered that the original site of this species is not far away from the type locality of *C.pubescens* with only a straight-line distance of about 100 km. The habitats of the two species are almost the same, and many of the common characteristics are shared by them, such as relatively short and slender culm with solid and initially white pubescent internodes, basal nodes with some short aerial roots, abaxially mainly glabrous culm leaf sheaths and relatively narrow foliage leaf blades. Therefore, we support that Wen (1994) reduced *C.solida* into a synonym of *C.pubescens*. However, it is found that both Wen and Yang incompletely recorded the height and thickness of their culms. In a superior habitat, this species can grow to 2.1 m tall and 1.1 cm in diameter, which can also be supported by Zhang (2009). *Chimonobambusaluzhiensis* was placed in *Qiongzhuea* when it was first published (Hsueh and Yi 1983), but it is characterized by culm usually with cylindrical internodes, basal nodes with protuberances or short aerial roots, persistent culm leaves with smaller culm leaf blades 2–9 mm long (Fig. 6), new shoots developed in October, which shows that it is just right to fall into C.sect.Chimonobambusa, so we support the treatment of Lan (1988), Ohrnberger (1990) and Wen (1994) to transfer it into *Chimonobambusa*.

## Supplementary Material

XML Treatment for Chimonobambusa farcta

## ﻿Additional information

### Author ORCIDs

Guang-Hui Lai https://orcid.org/0000-0003-0691-8165

Jin-Jun Yue https://orcid.org/0000-0001-9606-6394

### Data availability

All of the data that support the findings of this study are available in the main text.
